# Silencing of the *TRIM58* Gene by Aberrant Promoter Methylation is Associated with a Poor Patient Outcome and Promotes Cell Proliferation and Migration in Clear Cell Renal Cell Carcinoma

**DOI:** 10.3389/fmolb.2021.655126

**Published:** 2021-03-16

**Authors:** Ying Gan, Congcong Cao, Aolin Li, Haifeng Song, Guanyu Kuang, Binglei Ma, Quan Zhang, Qian Zhang

**Affiliations:** ^1^ Department of Urology, Peking University First Hospital and Institute of Urology, Peking University, Beijing, China; ^2^ National Urological Cancer Center, Beijing, China; ^3^ Beijing Key Laboratory of Urogenital Diseases (male) Molecular Diagnosis and Treatment Center, Beijing, China; ^4^ The Guangdong and Shenzhen Key Laboratory of Male Reproductive Medicine and Genetics, Peking University Shenzhen Hospital, Institute of Urology of Shenzhen PKU‐HKUST Medical Center, Shenzhen, China

**Keywords:** clear cell renal cell carcinoma, TRIM58, DNA methylation, engineered demethyltransferase, CRISPR-dCas9

## Abstract

To investigate the underlying molecular mechanism of tripartite motif-containing 58 (TRIM58) in the development of clear cell renal cell carcinoma (ccRCC), we explored TRIM58 expression and methylation in tumor tissues and the association with clinicopathological features and prognosis of tissue samples; Moreover, we examined the direct gene transcription of TRIM58-specific DNA demethyltransferase (TRIM58-TET1) by the CRISPR-dCas9 fused with the catalytic domain of TET1 and the biological functions in RCC cells. In this study, we demonstrate that TRIM58 is frequently downregulated by promoter methylation in ccRCC tissues, associated significantly with tumor nuclear grade and poor patient survival. TRIM58-TET1 directly induces demethylation of TRIM58 CpG islands, and activates TRIM58 transcription in RCC cell lines. Besides, DNA demethylation of TRIM58 by TRIM58-TET1 significantly inhibits cell proliferation and migration Overall, our results demonstrate that TRIM58 is inactivated by promoter methylation, associates with tumor nuclear grade and poor survival, and TRIM58 DNA demethylation could directly activate TRIM58 transcription and inhibit cell proliferation and migration in RCC cell lines.

## Introduction

Renal cell carcinoma (RCC) is one of the most common malignancies of the urinary system and accounts for approximately 2–3% of adult malignant solid tumors. The incidence of this disease has been steadily increasing in recent decades, partially because of increase in life expectancy and advance in medical imaging techniques (Ferlay et al., 2015; Bray et al., 2018). RCC lacks specific clinical manifestations, and approximately 20–30% of patients were reported to be initially diagnosed with distant metastasis (Alt et al., 2011). The response rate to targeted therapy and immunotherapy for metastatic patients is only approximately 30%. With the emergence of drug resistance, the vast majority of patients will eventually die from tumor progression (Linehan and Ricketts, 2014). Clear cell renal cell carcinoma (ccRCC) is the most common pathological subtype of RCC (accounting for 75–80%) (Moch, 2013). Therefore, it is of great significance to further investigate the molecular mechanism underlying ccRCC occurrence and development, which will benefit future research in discovering effective biomarkers and therapeutic targets.

Genome-wide epigenetic alterations are associated with human tumorigenesis. The silence of tumor suppressor genes (TSGs) by aberrant promoter methylation happens frequently and is recognized as a hallmark of the occurrence and development of cancers, including RCC (Sharma et al., 2010; Klutstein et al., 2016). Many critical TSGs have been reported to be inactivated by promoter methylation in RCC, such as APAF-1, RASSF1A, SFRP, CADM2, CDKN2A, DKK1, IRF8, and SOX7 (Morrissey et al., 2001; Christoph et al., 2006; Hirata et al., 2011; He et al., 2013; Ricketts et al., 2014; Zhang et al., 2014; Ricketts et al., 2018; Wang et al., 2019), among others. TRIM58 (tripartite motif-containing 58), a member of the TRIM family, which is located at 1q44, encodes a protein that exhibits E3 ubiquitin ligase activity. It was found to participate in a wide range of physiological processes, such as innate immunity, cell proliferation, and DNA damage repair, as well as many genetic diseases and cancers (Hatakeyama, 2011). Studies have revealed that the TRIM58 gene is hypermethylated and downregulated in a variety of malignancies including lung cancer, liver cancer, and pancreatic ductal adenocarcinoma (Tao et al., 2011; Kajiura et al., 2017; Xu et al., 2019). Moreover, TRIM58 protein can inhibit proliferation and invasion in gastrointestinal cancer cells, indicating the tumor-suppressive role in such cancers (Liu M. et al., 2018; Liu et al., 2020). However, the direct methylation–expression pattern and biological function of TRIM58 in ccRCC remains unclear.

To dissect the functional significance of DNA methylation events in human cancer, CRISPR–dCas9 has been used for targeted epigenome editing by fusion with epigenome modifying enzymes such as Dnmt3a (a DNA methyltransferase enzyme) and TET (catalyze active DNA demethylation) (Sasaki and Matsui, 2008; Morita et al., 2016; Vojta et al., 2016; Xu et al., 2016). A catalytically inactive dead Cas9 (dCas9)-based system fused to the catalytic domain of TET1 (TET1CD) that hydroxylates specific loci. This system has been designed to activate site-specific demethylation in the genome DNA (Yin and Xu, 2016; Gallego-Bartolome et al., 2018; Liu X. S. et al., 2018). Here, a TRIM58-specific DNA demethyltransferase (TRIM58-TET1) was used to directly activate TRIM58 transcription and the subsequent functional effects.

In this study, we investigated whether TRIM58 was a newly identified tumor suppressor gene with aberrant promoter methylation for ccRCC. To get this conclusion, we explored TRIM58 expression and methylation status in primary tumors, as well as its relationship with clinicopathological characteristics and survival. We also examined its tumor-suppressive functions by TRIM58-TET1 in RCC cells.

## Results

### TRIM58 was Frequently Silenced and Promoter Methylated in ccRCC Samples

**FIGURE 1:**
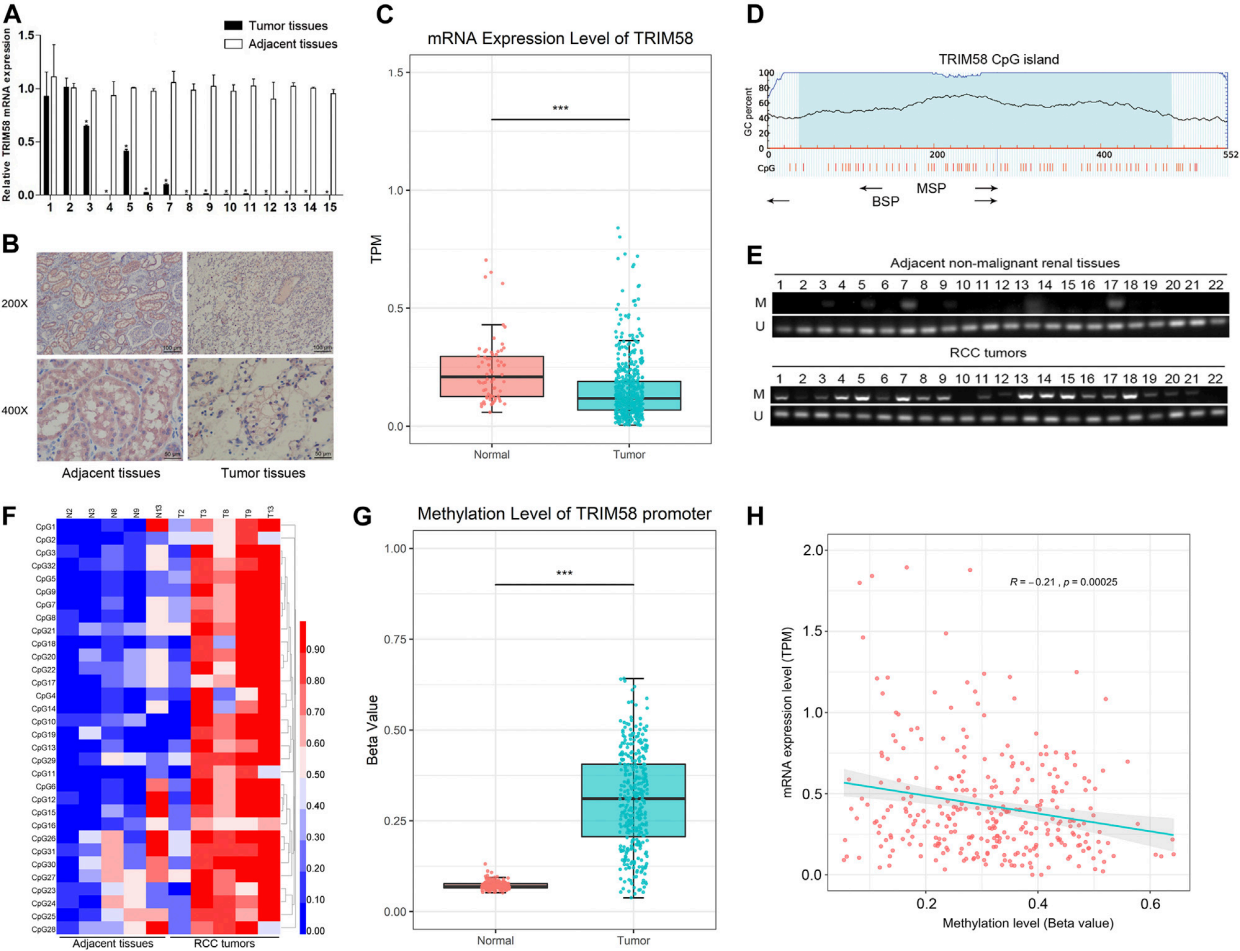
TRIM58 is frequently silenced and hypermethylated in ccRCC samples. **(A)** The relative mRNA transcription level of TRIM58 in 15 paired renal tumor tissues and the adjacent non-malignant renal tissues; **(B)** Representative IHC staining for TRIM58 in human ccRCC cancer **(right panels)** and adjacent normal renal tissues **(left panels)**; **(C)** mRNA transcription level of TRIM58 in KIRC from TCGA database; **(D)** Primers targeting TRIM58 promoter region designed for MSP and BGS; **(E)** MSP analysis in 92 ccRCC samples and 22 adjacent non-malignant renal tissues from our center; **(F)** BGS analysis on TRIM58 promoter 32 CpG sites of five ccRCC samples with paired adjacent tissues; **(G)** Methylation level of 317 KIRC patients from UALCAN; **(H)** Correlation analysis between the expression and methylation of TRIM58.

To evaluate TRIM58 expression in ccRCC tissues, the mRNA transcription level of TRIM58 was examined by qRT-PCR in ccRCC samples compared to that in adjacent non-malignant renal tissues from our center. Compared with the adjacent non-malignant renal tissues, TRIM58 was significantly decreased in 15 paired renal tumor tissues (*p* < 0.001, Figure 1A). TRIM58 protein expression levels were also decreased in ccRCC samples according to IHC, as compared to those in adjacent non-malignant renal tissue (Figure 1B). We next validated the expression pattern of TRIM58 in KIRC using the TCGA database. According to RNA-sequence data from 530 KIRC patients, KIRC exhibited significantly decreased expression of *TRIM58* mRNA compared to that in normal renal tissues (*p* < 0.001, Figure 1C).

It is generally known that DNA methylation in the promoter region may directly in-activate gene transcription. By exploring the UCSC Genome Browser, we found there is a CpG island near the TRIM58 promoter region. It is attractive that if there are any change of DNA methylation status of TRIM58 promoter region in ccRCC. TRIM58 methylation status was analyzed in 92 ccRCC samples and 22 adjacent non-malignant renal tissues from our center. Primers targeting this region were designed for MSP and BGS (Figure 1D). MSP analysis showed that methylation of the TRIM58 promoter could be detected in 80.4% (74/92) of ccRCC samples but in only 18.2% (4/22) of adjacent non-malignant renal tissues (*p* < 0.001, Figure 1E). As expected, BGS analysis of five ccRCC samples with paired adjacent non-malignant tissues demonstrated that TRIM58 methylation levels were greater in ccRCC tissues based on 32 CpG sites (Figure 1F). Meanwhile, we analyzed the methylation data of 317 KIRC patients from UALCAN and found TRIM58 promoter methylation level in KIRC was significantly higher than that in normal renal tissue (*p* < 0.001, Figure 1G). To investigate the relationship between the expression and promoter methylation of TRIM58, we performed correlation analysis. The results showed a significant negative correlation between mRNA expression and promoter methylation levels for this gene (R = −0.21, *p* < 0.001, Figure 1H).

### TRIM58 Hypermethylation and Low Expression was Associated With Clinicopathological Features and Poor Prognosis in ccRCC Samples

We evaluated the relationship between TRIM58 methylation and clinicopathological features of RCC patients from our center. According to their TRIM58 methylation status of tumor tissue samples, 92 KIRC patients were allocated into a methylated group and a non-methylated group, with their corresponding clinicopathological characteristics analyzed. Among the 92 patients, 63 were males and 29 were females, with an average age of 57.28 ± 12.42 years. We found that the TRIM58 promoter methylation was significantly associated with nuclear grade. Nuclear grade was higher in the methylated group compared to the unmethylated group (*p* = 0.02). Moreover, methylation of the TRIM58 promoter was not significantly associated with patient age, sex, and T stage (Table 1). UALCAN patients were then divided into high and low methylation groups according to the median TRIM58 methylation level. Chi-square test showed that the methylation of TRIM58 was significantly associated with T stage and nuclear grade in ccRCC; patients with a high methylation level had higher T stages (*p* < 0.001) and nuclear grades (*p* < 0.001, Table 1).

**TABLE 1:** Association of TRIM58 promotor methylation with clinicopathological features in ccRCC.

Features	PKUFH (n = 92)			TCGA (n = 317)		
	Methylated (%)	Unmethylated (%)	p	High (%)	Low (%)	*p*
Age			0.056			0.154
<65	145 (54.9%)	119 (45.1%)		67 (44.7%)	83 (55.3%)	
≥65	124 (46.6%)	142 (53.4)		88 (52.7%)	79 (47.3%)	
Gender			0.637			0.055
Male	172 (50%)	172 (50%)		107 (53.0%)	95 (47.0%)	
Female	97 (52.2)	89 (47.8%)		48 (41.7%)	67 (58.3%)	
T stage			0.142			<**0.001**
T1-T2	59 (77.6%)	17 (22.4%)		73 (36.5%)	127 (63.5%)	
T3-T4	15 (93.7%)	1 (6.2%)		82 (70.1%)	35 (29.9%)	
Nuclear grade			**0.020**			<**0.001**
G1-G2	56 (75.7%)	18 (24.3%)		52 (35.6%)	94 (64.4%)	
G3-G4	18 (100%)	0 (0%)		103 (60.2%)	68 (39.8%)	

Bold values indicate statistical significance.

PKUFH, Peking University First Hospital; TCGA, The Cancer Genome Atlas.

To further explore the correlation between TRIM58 DNA methylation inactivation and the prognosis of KIRC patients, the median mRNA expression level of TRIM58 was separately set as the cut-off value using the TCGA database. Survival analysis indicated significant differences between high and low expression groups in terms of both overall survival (OS) and disease-free survival (DFS); patients with high *TRIM58* expression had a longer OS (*p* = 0.025, Supplementary Figure S1A) and DFS (*p* = 0.02, Supplementary Figure S1B). In addition, patients from UALCAN with a high TRIM58 methylation level had significant worse OS (*p* = 0.014, Supplementary Figure S1C) and DFS (*p* < 0.001, Supplementary Figure S1D) than patients with low methylation. These results indicate that TRIM58 methylation inactivation is correlated with the poor prognosis of KIRC.

### Methylation of TRIM58 Promoter Correlated With Its Downregulation in RCC Cell Lines

**FIGURE 2:**
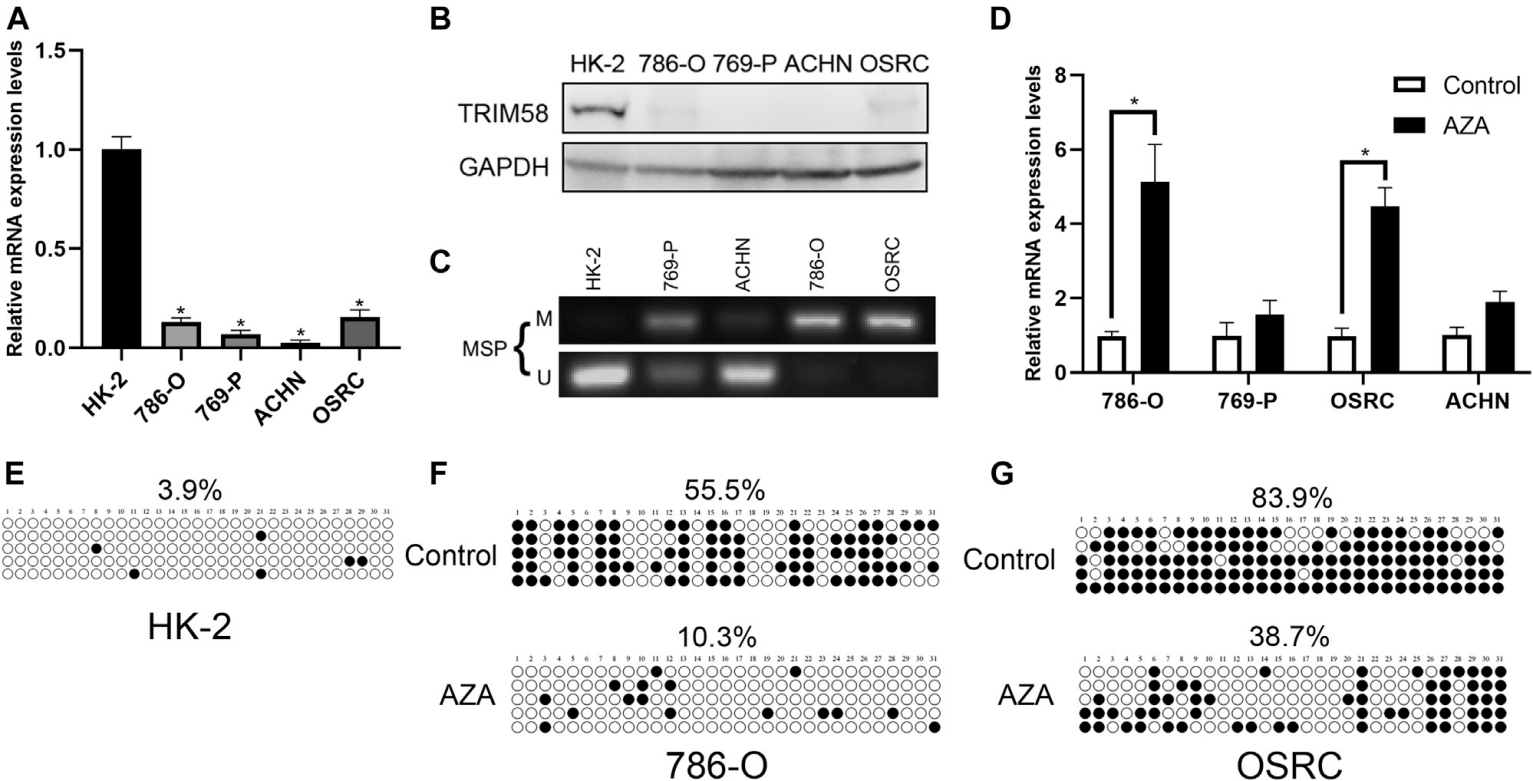
TRIM58 methylation correlates with its downregulation in RCC cell lines. **(A**, **B)** TRIM58 expression in RCC cell lines (786-O, 769-P, ACHN and OSRC) and HK-2 normal cells using qRT-PCR **(A)** and Western Blot **(B)**; **(C)** MSP for methylation status of the TRIM58 promoter; **(D)** TRIM58 mRNA expression level after treating with the demethylation drug 5-AZA; **(E)** Methylation pattern of TRIM58 in HK-2 cells; **(F**, **G)** TRIM58 promoter CpG site methylation after treating with 5-AZA in 786-O and OS-RC-2 cells.

To evaluate the expression level of TRIM58 in RCC cell lines, we examined mRNA and protein expression in RCC cell lines (786-O, 769-P, ACHN and OS-RC-2). The TRIM58 expression was significantly reduced and even silenced, when compared to that in HK-2 normal human kidney proximal tubular epithelial cells using qRT-PCR (Figure 2A) and Western Blot (Figure 2B). We then examined the methylation status of the TRIM58 promoter in those cells. MSP results showed that the RCC cell lines, especially 786-O and OS-RC-2, exhibited DNA hypermethylation in the promoter region of TRIM58 compared to HK-2 cells (Figure 2C).

To determine whether TRIM58 expression was directly repressed by DNA methylation, we examined the TRIM58 mRNA expression level and DNA methylation status in RCC cell lines after treating them with the demethylation drug 5-AZA. The TRIM58 mRNA levels were restored after the treatments (Figure 2D), accompanied with a decrease of TRIM58 promoter CpG site methylation from ∼55.5% to ∼10.3% in 786-O, and from ∼83.9% to ∼38.7% in OS-RC-2, respectively (Figures 2E–G). These results suggested that TRIM58 promoter methylation mediates its decreased expression in RCC cells.

### TRIM58 DNA Demethylation Directly Activated Gene Transcription

**FIGURE 3:**
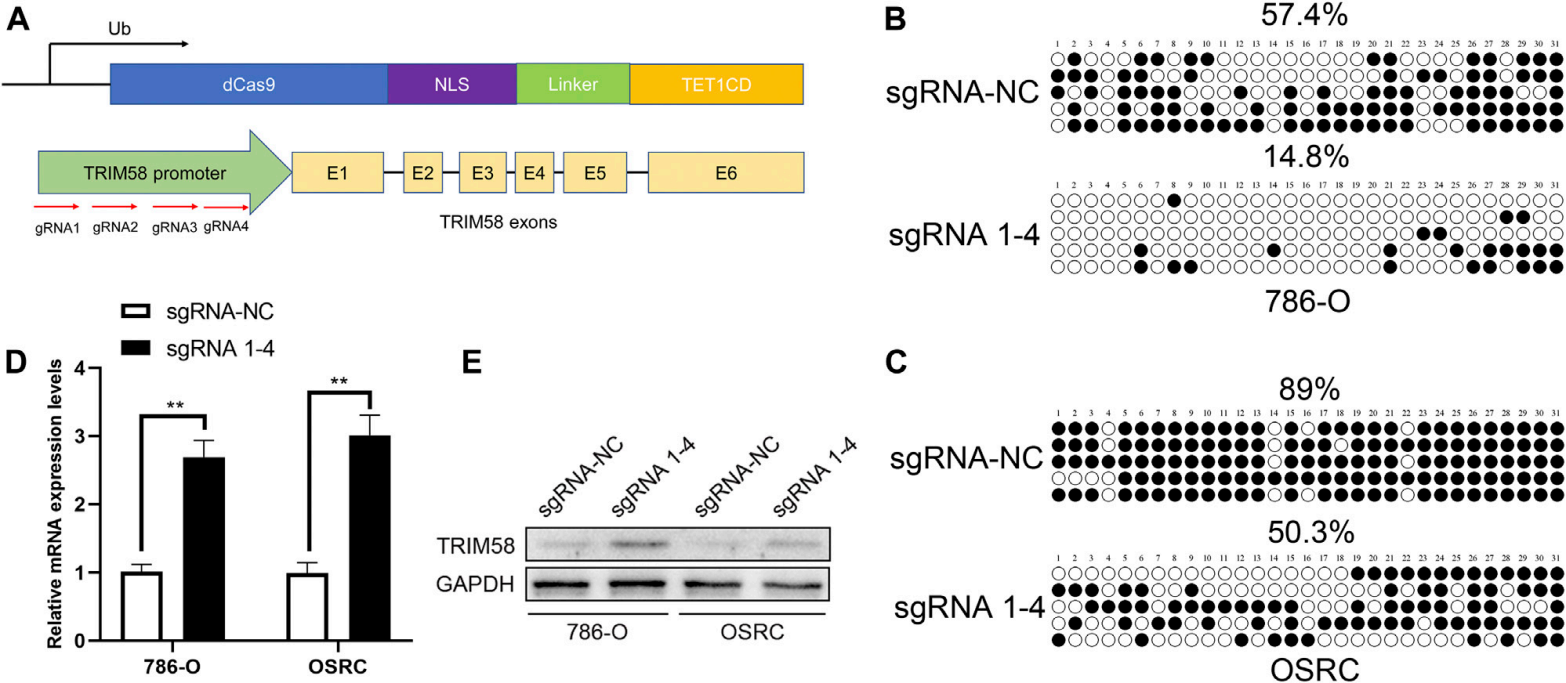
TRIM58 targeted demethylation directly activates gene transcription. **(A)** Contruction of TRIM58-TET1 using CRISPR-dCas9 systems with 4 gRNAs targeting TRIM58 promoter region and the catalytic domain of DNA hydroxymethylase; **(B**, **C)** Bisulfite clone-sequencing results from 786-O and OS-RC-2 cells transiently transfected with TRIM58-TET1; **(D**, **E)** TRIM58 gene transcription **(D)** and protein expression **(E)** following TRIM58-TET1 transfection.

To explore whether DNA demethylation is essential for re-activation of TRIM58, TRIM58 promoter-specific DNA demethyltransferase (TRIM58-TET1) leading by four guide RNAs (sgRNA1-4) using CRISPR-dCas9 systems (Figure 3A) was transfected transiently into 786-O and OS-RC-2 RCC cell lines. Then, methylation of CpG islands within the TRIM58 promoter region was detected using bisulfite-sequencing. As expected, TRIM58-TET1 induced TRIM58 DNA demethylation in both 786-O and OS-RC-2 cells (Figures 3B,C) and significantly activated TRIM58 gene transcription and protein expression (Figures 3D,E). Taken together, these results indicated that TRIM58 promoter region demethylation could directly activate gene transcription and expression.

### TRIM58 DNA Demethylation Suppressed Cell Proliferation and Migration in RCC Cells

**FIGURE 4:**
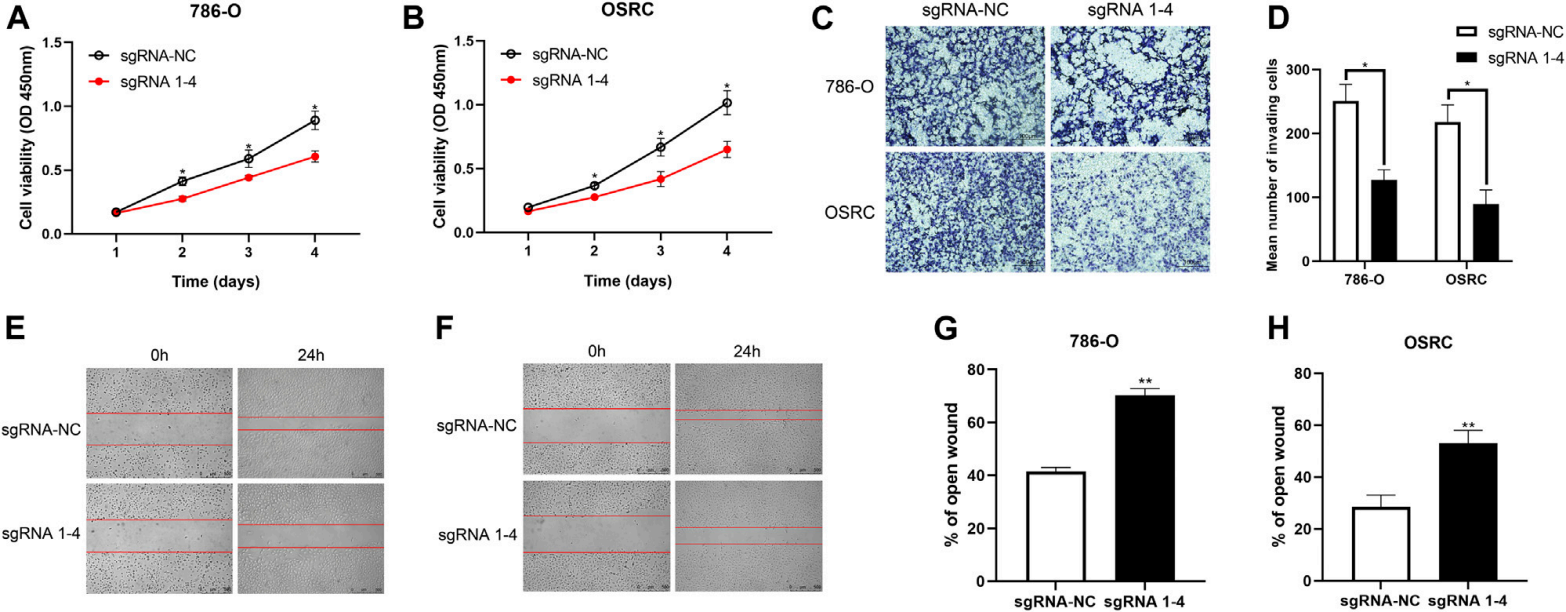
Proliferation and migration assays carried out with RCC cells transient transfected with TRIM58-TET1 vector. **(A**, **B)** CCK-8 assay for cell proliferation of TRIM58-TET1 transient transfected 786-O and OS-RC-2 cells; **(C**, **D)** Transwell assays for the migration ability of 786-O and OS-RC-2 cells; **(E–H)** The wound-healing assays for TRIM58 demethylation in both RCC cells.

To characterize the biological behaviors of cancer cells following TRIM58-specific activation by DNA demethylation, we determined whether TRIM58 demethylation inhibited RCC cell proliferation. As shown in Figures 4A,B, we found that TRIM58 demethylation in 786-O and OS-RC-2 cells significantly inhibited cell proliferation by CCK-8 assay. Next, we evaluated the migration ability of TRIM58 demethylation in RCC cell lines by transwell migration assays and wound healing. The migration ability of 786-O and OS-RC-2 cells was significantly decreased following TRIM58 demethylation (Figures 4C,D). The wound-healing assays showed that TRIM58 demethylation in both RCC cells closed the wound much slower than controls (Figures 4E–H). Taken together, these results implied that TRIM58 DNA demethylation reduced the proliferation and migration phenotypes of RCC cells.

## Discussion

Methylation-mediated silencing of tumor-suppressor genes is a critical event in the occurrence and development of various malignancies, including ccRCC(Malouf et al., 2016; Morris and Latif, 2017; Joosten et al., 2018). In the previous series of research, we identified some tumor-specifically methylated genes in RCC, such as DLC1, DLEC1, IRF8, ADAMTS18 (Zhang et al., 2007; Zhang et al., 2010; Zhang et al., 2014). TRIM58, a member of the TRIM family, has been reported to play a tumor suppressive role and to be regulated by DNA methylation in lung cancer, colorectal cancer, and gastric cancer (Kajiura et al., 2017; Liu M. et al., 2018; Liu et al., 2020). However, its DNA methylation status and biological function in ccRCC remained unclear. In the present study, we preliminarily verified that TRIM58 is silenced and hypermethylated in ccRCC based on the TCGA database. The methylation level of TRIM58 was strongly associated with nuclear grade in both our samples and database, as well as with tumor stage in database. Although patients with higher T stages had higher methylation rates of TRIM58 than patients with lower T stages in our center, no statistical difference was found between them. We speculated that this was because of the small sample size of high T stage patients. In addition, ccRCC patients with high TRIM58 expression and hypomethylation levels of TRIM58 have better OS and DFS.

*In vitro*, we confirmed the methylation of TRIM58 in several RCC cell lines. RCC cells with low TRIM58 expression were found to be hypermethylated and the demethylation drug 5-AZA upregulated the expression of TRIM58, suggesting that the low expression of TRIM58 in ccRCC was probably induced by promoter methylation. We found TRIM58 was frequently inactivated and methylated in RCC cell lines and in primary RCC tissues. Thus, we investigated whether TRIM58 demethylation is capable of activating transcription. To target specific epigenetic alterations in cancer cells, we selected the CRISPR-dCas9 system. CRISPR-dCas9 was derived from the CRISPR-Cas9 system, and has been used in many fields such as gene regulation, epigenetic regulation and high throughput screening. dCas9 lacks nuclease activity but maintains its ability to bind both the sgRNA and targeted DNA. Several groups have developed modified versions of Cas9 for applications that go beyond genome editing (Bikard et al., 2013; Chavez et al., 2015; Kardooni et al., 2018). Here, we used dCas9-TET1 and gRNAs targeting the TRIM58 gene promoter to induce DNA demethylaton and activate transcription.

In lung squamous cell carcinoma, TRIM58 methylation is associated with eight prognostic genes, and may be used as a potential prognostic biomarker (Zhang et al., 2018). In gastric cancer, TRIM58 may function as a tumor suppressor and potentially suppress the tumor growth (Liu et al., 2020). In early-stage lung adenocarcinoma, TRIM58 was robustly silenced by hypermethylation and the restoration of TRIM58 expression in cell lines inhibited cell growth *in vitro* and *in vivo* (Kajiura et al., 2017). In the present study, we have provided evidence to demonstrate that TRIM58-TET1 induced demethylation could directly inhibit proliferation and migration of cancer cells *in vitro*. These facts strongly implicate the inactivation of TRIM58 by DNA methylation as a possible promoter of proliferation and migration in RCC.

## Materials and Methods

### Patients and Tissue Specimen Collection

92 primary clear cell renal cell carcinoma (ccRCC) samples and 22 adjacent non-malignant renal tissues with patients’ informed consent were obtained from the Urology Department of Peking University First Hospital (PKUFH), Beijing, China. This study followed the Helsinki declaration and was approved by the Institutional Ethical Review Board of PKUFH. Samples were collected immediately in the operating room after surgical removal and were stored in liquid nitrogen after rapid freezing in liquid nitrogen. The pathological diagnosis was made by professional urological pathologists.

Further, mRNA expression and clinical information for 530 kidney renal clear cell carcinoma (KIRC) patients with available RNA-sequence data were downloaded from The Cancer Genome Atlas (TCGA). Methylation data (Illumina Human Methylation 450K BeadChip) of 317 KIRC patients were downloaded from UALCAN (http://ualcan.path.uab.edu/).

### Cell Lines Culture and Demethylation Treatment

RCC cell lines (786-O, 769-P, ACHN and OS-RC-2) were used in this study. HK-2 human kidney proximal tubular epithelial cells were used as normal controls. These cell lines were purchased from the American Type Culture Collection (ATCC, Manassas, VA, United States) and National Infrastructure of Cell Line Resource, China. Cell lines were routinely cultured in RPMI 1640 or DMEM, which was supplemented with 10% fetal bovine serum (Invitrogen, Carlsbad, CA, United States) and incubated in a 5% CO_2_ environment at 37°C. These cells were split to a low density (30% confluence) for 12 h before drug treatment, then incubated with 5-aza-2′-deoxycytidine (5-Aza; Sigma, St. Louis, MO, United States) at a concentration of 10 μM in the optional medium, which was exchanged every 24 h. After 5-Aza treatment for 72 h, the cells were harvested for further analysis.

### Construction of Vectors and Transfection

The sequences of the TRIM58 gene promoter region containing the CpG islands were extracted using the UCSC genome browser. The CRISPR dCas9 plasmid dCas9-Tet1CD (#84475) and pgRNA-humanized (#44248) were purchased from Addgene. Four gRNAs that target the TRIM58 promoter were cloned into pgRNA-humanized and listed in Table 2. All designed gRNAs were subjected to MEGABLAST (https://blast.ncbi.nlm.nih.gov/Blast.cgi) to avoid mismatch to unexpected genes in the human genome.

**TABLE 2:** Primers for RT-qPCR, MSP, BGS and sequence of gRNAs.

Target	Sequence (5′-3′)	Application
TRIM58	F: GGT​GTG​TTT​GGA​TTT​TTT​GTA​GGA​G	RT-qPCR
	R: CCA​CAA​CCA​AAA​CAA​AAA​AAC​C	
GAPDH	F: GGA​GCG​AGA​TCC​CTC​CAA​AAT	RT-qPCR
	R: GGC​TGT​TGT​CAT​ACT​TCT​CAT​GG	
TRIM58	M-F: CGT​TTA​CGT​TTG​TTC​GTA​GTG​TC	MSP
	M-U: CAA​AAA​CGA​CTC​AAA​TCC​TCG	
TRIM58	U-F: TGT​TTA​TGT​TTG​TTT​GTA​GTG​TTG	MSP
	U-R: CAA​AAA​CAA​CTC​AAA​TCC​TCA​CC	
TRIM58	F: GAG​GAG​GGA​TTT​TAG​TTA​GAA​ATG​TTT	BGS
	R: ACT​CCT​ACA​AAA​AAT​CCA​AAC​ACA​C	
TRIM58-sgRNA-1	F: TTG​GGT​ACG​TTT​GTT​CGT​AGT​GTC​GGG​GC	dcas9-TET1CD
	R: GAA​CAA​CCC​ATG​CAA​ACA​AGC​ATC​ACA​GC CCCGAGCT	
TRIM58-sgRNA-2	F: TTG​GGA​GTC​GGT​TAG​CGT​GGA​TTG​GGG​C	dcas9-TET1CD
	R: GAA​CAA​CCC​TCA​GCC​AAT​CGC​ACC​TAA​C CCCGAGCT	
TRIM58-sgRNA-3	F: TTG​GGC​CTC​GGG​CTT​TCG​CCC​CAA​CGG​GC	dcas9-TET1CD
	R: GAA​CAA​CCC​CGG​TTG​GGG​CGA​AAG​CCC​GA CCCGAGCT	
TRIM58-sgRNA-4	F: TTG​GGC​GGG​CCT​GGT​GGA​GAG​CGT​GGG​GC	dcas9-TET1CD
	R: GAA​CAA​CCC​CAC​GCT​CTC​CAC​CAG​GCC​CG CCCGAGCT	
TRIM58-sgRNA-NC	F:TTGGGGTAATGCCTGGCTTGTCGACGCATAGTCTGGGGC	dcas9-TET1CD
	R:GAACAACCCCAGACTATGCGTCGACAAGCCAGGCATTACCCCGAGCT	

### DNA and RNA Extraction

Total RNA and genomic DNA of primary RCC tissues and cell lines were extracted using an RNA-easy Isolation Reagent (Vazyme Biotech, Nanjing, China) and TIANamp Genomic DNA Kits (TIANGEN, Shanghai, China) according to their instructions, respectively, as previously described (Zhang et al., 2014; Wang et al., 2019).

### Bisulfite Treatment and Promoter Methylation Analysis

The bisulfite modification of genomic DNA was performed using the EZ DNA Methylation-Gold™ Kit (Zymo Research, Menlo Park, CA, United States). Methylation specific PCR (MSP) and bisulfite genomic sequencing (BGS) were used to analyze methylation status of TRIM58 promoter region. The primers for TRIM58 used for MSP and BGS were listed in Table 2. For BGS, PCR products were subcloned into the fast-T1 clone vector (Vazyme Biotech, Nanjing, China) and 8–10 colonies were randomly selected and sequenced.

### Quantitative RT-PCR, Western Blot and Immunohistochemistry Staining of TRIM58 Expression

cDNA was synthesized using HiScript III RT SuperMix for qPCR (Vazyme Biotech, Nanjing, China). qRT-PCR was performed using spectrophotometry (ABI Prism 7500TM instrument, Applied Biosystems) with Universal SYBR Green qPCR Master Mix (Vazyme Biotech, Nanjing, China). Glyceraldehyde 3-phosphate dehydrogenase (GAPDH) was used as reference gene. Primers used for TRIM58 and GAPDH qRT-PCR were listed in Table 2.

Total protein was extracted by KeyGEN Bio TECH protein extraction kit (KGP1100) and separated on 10% SDS-PAGE and transferred onto nitrocellulose membrane. After blocking, blots were immunostained with primary antibodies and secondary antibodies respectively as previously described (Wang et al., 2017). The antibodies were as follows: TRIM58 (ab254786, Abcam, 1:500); GAPDH (0494-1-AP, proteinteach, 1:10000).

Immunohistochemistry staining was performed using a primary antibody of TRIM58 (ab254786, Abcam) at a 1:300 dilution following a protocol described previously (Liu et al., 2015). All photographs were taken randomly and measured using Image Pro Plus (Media Cybernetics, Rockville, MD, United States).

### Wound-Healing Assay

The cell motility was assessed by scratch wound healing assay. 786-O and OS-RC-2 cells (2–3×10^6^ per well) were plated in a 6-well plate for 1 day and then transfected with vectors for 24 h. The cell layers were washed with PBS after carefully scratching by sterile tips. After incubation for 0 and 24 h, photos were taken. The assays were performed in triplicate.

### Transwell Migration Assay

The 786-O and OS-RC-2 cells suspended in 150 uL serum-free medium (2×10^5^ cells/mL) were placed on the upper layer of a cell permeable membrane. Following another 24–48 h incubation, the cells migrated through the membrane were stained with 1% Crystal Violet and counted.

### Statistical Analysis

When comparing two groups of measurement data, *t* test was used for data conforming to normal distribution, whereas a Wilcoxon test was used for data not conforming to normal distribution, and the measurement data were expressed as the mean ± standard deviation (SD). A Chi-square test was used to analyze comparisons between groups for enumeration data. Pearson correlation analysis was used to investigate the relationship between mRNA expression levels and methylation levels of TRIM58. The Kaplan–Meier method was used for survival analysis, and a log-rank test was applied for comparations between groups. R packages used in this study included “GDCRNATools,” “clusterProfiler,” “org.Hs.eg.db,” “tidyr,” “dplyr,” “ggplot2,” “ggsignif,” “survival,” and “survimier.” Annotation gene sets used in GSEA were hallmark gene sets from the Molecular Signatures Database (MSigDB). All statistical analyses were performed and visualized using RStudio (Version1.2.1335, Boston, MA, United States), GSEA (Version4.0, UC San Diego and Broad Institute, United States) 23, Medcalc (Version16.8, Ostend, Belgium), and GraphPad Prism (Version 8.0, GraphPad, Inc., La Jolla, CA, United States). A two-tailed *p* < 0.05 was considered statistically significant.

## Data Availability

The original contributions presented in the study are included in the article/Supplementary Material, further inquiries can be directed to the corresponding author.
