# Supplementary material for: Silencing of the TRIM58 Gene by Aberrant Promoter Methylation is Associated with a Poor Patient Outcome and Promotes Cell Proliferation and Migration in Clear Cell Renal Cell Carcinoma
Source: Front Mol Biosci. 2021 Mar 16;8:655126. doi: 10.3389/fmolb.2021.655126 (PMC8012909; doi:10.3389/fmolb.2021.655126)
Supplement: Supplementary file 1 [file table1.docx]

Table 1 Primers for RT-qPCR, MSP, BGS and sequence of gRNAs

| TARGET | SEQUENCE (5’-3’) | APPLICATION |
| --- | --- | --- |
| TRIM58 | F: GGTGTGTTTGGATTTTTTGTAGGAG | RT-qPCR |
|  | R: CCACAACCAAAACAAAAAAACC |  |
| GAPDH | F: GGAGCGAGATCCCTCCAAAAT | RT-qPCR |
|  | R: GGCTGTTGTCATACTTCTCATGG |  |
| TRIM58 | M-F: CGTTTACGTTTGTTCGTAGTGTC | MSP |
|  | M-U: CAAAAACGACTCAAATCCTCG |  |
| TRIM58 | U-F: TGTTTATGTTTGTTTGTAGTGTTG | MSP |
|  | U-R: CAAAAACAACTCAAATCCTCACC |  |
| TRIM58 | F: GAGGAGGGATTTTAGTTAGAAATGTTT | BGS |
|  | R: ACTCCTACAAAAAATCCAAACACAC |  |
| TRIM58-sgRNA-1 | F: TTGGGTACGTTTGTTCGTAGTGTCGGGGC | dcas9-TET1CD |
|  | R: GAACAACCCATGCAAACAAGCATCACAGC CCCGAGCT |  |
| TRIM58-sgRNA-2 | F: TTGGGAGTCGGTTAGCGTGGATTGGGGC | dcas9-TET1CD |
|  | R: GAACAACCCTCAGCCAATCGCACCTAAC CCCGAGCT |  |
| TRIM58-sgRNA-3 | F: TTGGGCCTCGGGCTTTCGCCCCAACGGGC | dcas9-TET1CD |
|  | R: GAACAACCCCGGTTGGGGCGAAAGCCCGA CCCGAGCT |  |
| TRIM58-sgRNA-4 | F: TTGGGCGGGCCTGGTGGAGAGCGTGGGGC | dcas9-TET1CD |
|  | R: GAACAACCCCACGCTCTCCACCAGGCCCG CCCGAGCT |  |
| TRIM58-sgRNA-NC | F:TTGGGGTAATGCCTGGCTTGTCGACGCATAGTCTGGGGC  R:GAACAACCCCAGACTATGCGTCGACAAGCCAGGCATTACCCCGAGCT | dcas9-TET1CD |
|  |  |  |
