# Supplementary material for: Silencing of the TRIM58 Gene by Aberrant Promoter Methylation is Associated with a Poor Patient Outcome and Promotes Cell Proliferation and Migration in Clear Cell Renal Cell Carcinoma
Source: Front Mol Biosci. 2021 Mar 16;8:655126. doi: 10.3389/fmolb.2021.655126 (PMC8012909; doi:10.3389/fmolb.2021.655126)
Supplement: Supplementary file 2 [file table2.docx]

**Table2. Association of TRIM58 promotor methylation with clinicopathological features in ccRCC**

| Features | PKUFH (n=92) | | | TCGA (n=317) | | |
| --- | --- | --- | --- | --- | --- | --- |
|  | Methylated (%) | Unmethylated (%) | p | High (%) | Low (%) | *p* |
| Age |  |  | 0.056 |  |  | 0.154 |
| <65 | 145(54.9%) | 119(45.1%) |  | 67(44.7%) | 83(55.3%) |  |
| ≥65 | 124(46.6%) | 142(53.4) |  | 88(52.7%) | 79(47.3%) |  |
| Gender |  |  | 0.637 |  |  | 0.055 |
| Male | 172(50%) | 172(50%) |  | 107(53.0%) | 95(47.0%) |  |
| Female | 97(52.2) | 89(47.8%) |  | 48(41.7%) | 67(58.3%) |  |
| T stage |  |  | 0.142 |  |  | **<0.001** |
| T1-T2 | 59(77.6%) | 17(22.4%) |  | 73(36.5%) | 127(63.5%) |  |
| T3-T4 | 15(93.7%) | 1(6.2%) |  | 82(70.1%) | 35(29.9%) |  |
| Nuclear grade |  |  | **0.020** |  |  | **<0.001** |
| G1-G2 | 56(75.7%) | 18(24.3%) |  | 52(35.6%) | 94(64.4%) |  |
| G3-G4 | 18(100%) | 0(0%) |  | 103(60.2%) | 68(39.8%) |  |

Bold values indicate statistical significance.

**Abbreviations**: **PKUFH**, Peking University First Hospital; **TCGA**,The Cancer Genome Atlas.
