# Supplementary material for: Silencing of the TRIM58 Gene by Aberrant Promoter Methylation is Associated with a Poor Patient Outcome and Promotes Cell Proliferation and Migration in Clear Cell Renal Cell Carcinoma
Source: Front Mol Biosci. 2021 Mar 16;8:655126. doi: 10.3389/fmolb.2021.655126 (PMC8012909; doi:10.3389/fmolb.2021.655126)
Supplement: Supplementary file 3 [file datasheet1.pdf]

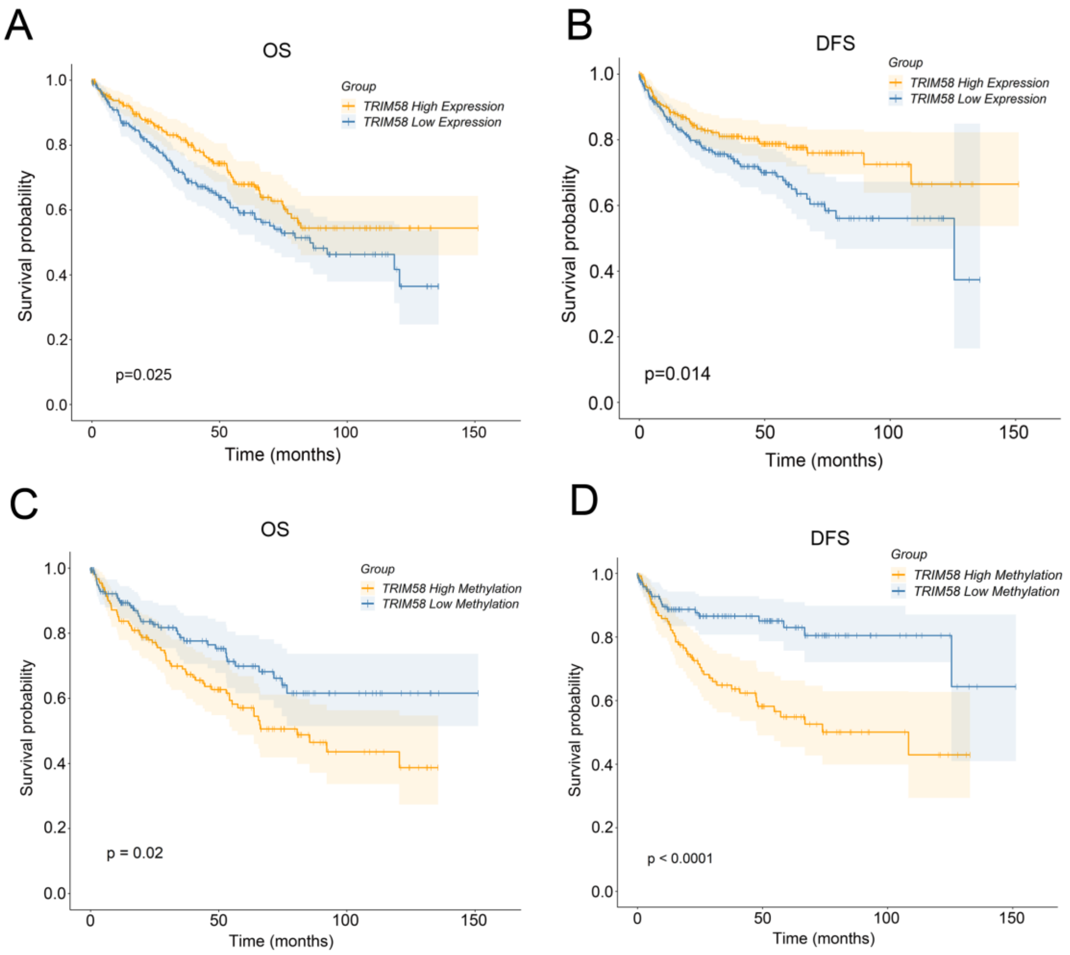

Figure S1 TRIM58 methylation inactivation is correlated with the poor prognosis of KIRC in database.

(A, B) Overall survival (OS) and disease-free survival (DFS) analysis for high and low TRIM58 expression groups in TCGA database; (C, D) OS and DFS analysis for TRIM58 hypermethylation and hypomethylation of patients from UALCAN database.
